# Involvement of p38 MAPK and MAPKAPK2 in promoting cell death and the inflammatory response to ischemic stress associated with necrotic glioblastoma

**DOI:** 10.1038/s41419-025-07335-3

**Published:** 2025-01-14

**Authors:** Soo Yeon Kim, Miaolu Tang, Stephen Y. Chih, Jessica Sallavanti, Yan Gao, Zhiqiang Qiu, Hong-Gang Wang, Wei Li

**Affiliations:** 1 Division of Hematology and Oncology, Department of Pediatrics, Penn State College of Medicine, Hershey, PA USA; 2 Medical Scientist Training Program, Penn State College of Medicine, Hershey, PA USA; 3 Penn State Cancer Institute, Penn State College of Medicine, Hershey, PA USA; 4 Department of Pharmacology, Penn State College of Medicine, Hershey, PA USA; 5 Department of Biochemistry and Molecular Biology, Penn State College of Medicine, Hershey, PA USA

**Keywords:** CNS cancer, Cell death, Stress signalling, Cancer metabolism

## Abstract

The association of necrosis in tumors with poor prognosis implies a potential tumor-promoting role. However, the mechanisms underlying cell death in this context and how damaged tissue contributes to tumor progression remain unclear. Here, we identified p38 mitogen-activated protein kinases (p38 MAPK, a.k.a. p38) as a key player in promoting cell death and the inflammatory response to ischemic stress associated with necrotic tumors. We found that glioblastoma (GBM) cells expressing patient-derived Kirsten rat sarcoma (KRAS) or phosphoinositide-3-kinase (PI3K) active mutants showed enhanced cell death under ischemia-mimetic conditions in vitro and were more likely to develop into necrotic tumors in vivo. Cell death in both settings depended on p38, which is also required for tumor progression driven by KRAS or PI3K. Under ischemia-mimetic conditions, GBM cells undergo reactive oxygen species (ROS)-dependent cell death. Gene expression in these cells recapitulated multiple features observed in peri-necrotic tumors from patient GBM. Further studies showed the involvement of a positive feedback loop between the p38-MAPK-activated protein kinase 2 (MAPKAPK2, a.k.a. MK2) signaling axis and the unfolded protein response signaling components activating transcription factor 4 (ATF4) and inositol-requiring enzyme 1 (IRE1α) in driving ischemic tumor cell death. This signaling cascade was further potentiated by RAS or PI3K activation under ischemic conditions, contributing to the inflammatory gene expression response. Therefore, our study suggests that p38 could be targeted to relieve the inflammatory response in necrotic tumors and inhibit GBM progression.

## Introduction

Necrosis is commonly observed in advanced solid tumors and is associated with poor prognosis. Necrotic tumor cells may promote tumor progression and resistance to various therapies by altering the immune system, creating chronic inflammation and immunosuppressive microenvironment [1, 2]. Therefore, developing strategies to mitigate the deleterious effect of tumor necrosis is of significant clinical importance.

Glioblastoma (GBM) is the most common and aggressive primary brain malignancy in adults. Similar to other cancers, necrosis is a diagnostic hallmark in GBM. However, how tumor cells undergo necrosis during GBM development remains incompletely understood. It is commonly accepted that ischemia, characterized by insufficient blood supply leading to oxygen and glucose deprivation, triggers metabolic stress in tumor cells, ultimately resulting in necrosis [3]. Consistent with this notion, GBM cells cultured under hypoxic and partially glucose-deprived conditions undergo necrotic cell death [4]. In addition to these metabolic cues, certain genetic features associated with GBM, such as activated Rat sarcoma (RAS) and Protein Kinase B (AKT) pathways, can affect cell viability and contribute to GBM necrosis [3]. Receptor tyrosine kinases (RTK)-RAS-phosphoinositide 3-kinases (PI3K) signaling is the most frequently (90%) activated signaling network in GBM [5, 6]. Activation of this signaling promotes cell growth, survival, and metabolism, contributing to its tumor-promoting roles. Interestingly, activating this pathway may also interfere with tumor cells’ adaptation to ischemic conditions. Under hypoxic and glucose deprivation conditions, ATP depletion and cell death can be delayed when epidermal growth factor receptor (EGFR) is inhibited [7]. Similarly, inhibition of the mammalian target of rapamycin (mTOR) or the combined inhibition of PI3K and extracellular signal-regulated kinase 1 and 2 (ERK1/2) has been shown to protect GBM cells from ischemic stress [7, 8]. Conversely, the expression of EGFRvIII, an active EGFR mutant, or the activation of mTOR complex 1 (mTORC1) through silencing the expression of its inhibitor tuberous sclerosis 2 (TSC2) exacerbates ATP depletion and increases the sensitivity of GBM cells to hypoxia-induced cell death [9, 10]. These findings suggested that the RTK-RAS-PI3K signaling network may regulate the response of tumor cells to the ischemic microenvironment in GBM. However, the exact downstream effectors and mechanisms through which these pathways influence cell fate under ischemic conditions remain to be fully elucidated.

p38 kinases, members of the mitogen-activated protein kinase (MAPK) family, are activated by various environmental stresses and inflammatory signals through the MAP3K-MAP2K kinase cascade [11]. Within this signaling pathway, p38 phosphorylates multiple substrate proteins, with MAPK-activated protein kinase 2 (MAPKAPK2, a.k.a. MK2) being a well-characterized target. As a stress-activated kinase, p38 contributes to cell survival by inducing cell cycle arrest, promoting DNA repair, and mitigating oxidative stress. This protective role is crucial in maintaining cellular integrity under adverse conditions. However, the role of p38 is complex, as sustained activation of p38 can lead to cell death. This occurs through various mechanisms, such as the phosphorylation and inhibition of the nuclear RNase III enzyme Drosha, which is essential for microRNA processing and regulation of gene expression [12]. Additionally, p38 can phosphorylate the Hippo pathway TEA Domain Transcription Factor (TEAD), leading to the disruption of cell proliferation and survival signals [13]. Another pathway involves the induction of cellular metabolic changes through MK2, which can shift cellular metabolism towards apoptosis under prolonged stress conditions [14]. p38 also plays a significant role in regulating inflammatory cytokine production in immune cells and other cell types. This regulation occurs through activating inflammatory regulators such as Nuclear factor kappa-B (NFkB) and MK2 [11]. Given the dual functionality of p38 in cell death and inflammation, it is important to explore whether p38 plays similar roles in tumor necrosis and the associated immune responses.

In this study, we investigated the response of GBM cells to ischemia using in vitro tissue culture models, orthotopic xenograft mouse models, and spatial gene expression profiles from patient GBM. Our results demonstrated that GBM cells expressing patient-derived active KRAS or PI3K mutants exhibited increased cell death under ischemia-mimetic conditions in vitro and were more prone to developing necrosis in vivo. The cell death in both scenarios depended on p38, which is also essential for tumor development driven by KRAS or PI3K. Further studies in vitro and using the patient GBM gene expression datasets suggest that the p38-MK2 signaling and the unfolded protein response (UPR) formed a positive feedback loop to amplify the effect of ischemia and promote tumor progression by driving the inflammatory response within necrotic tumors.

## Results

### Activation of RAS and PI3K enhances cell death in an ischemia-mimetic condition

**Fig. 1:**
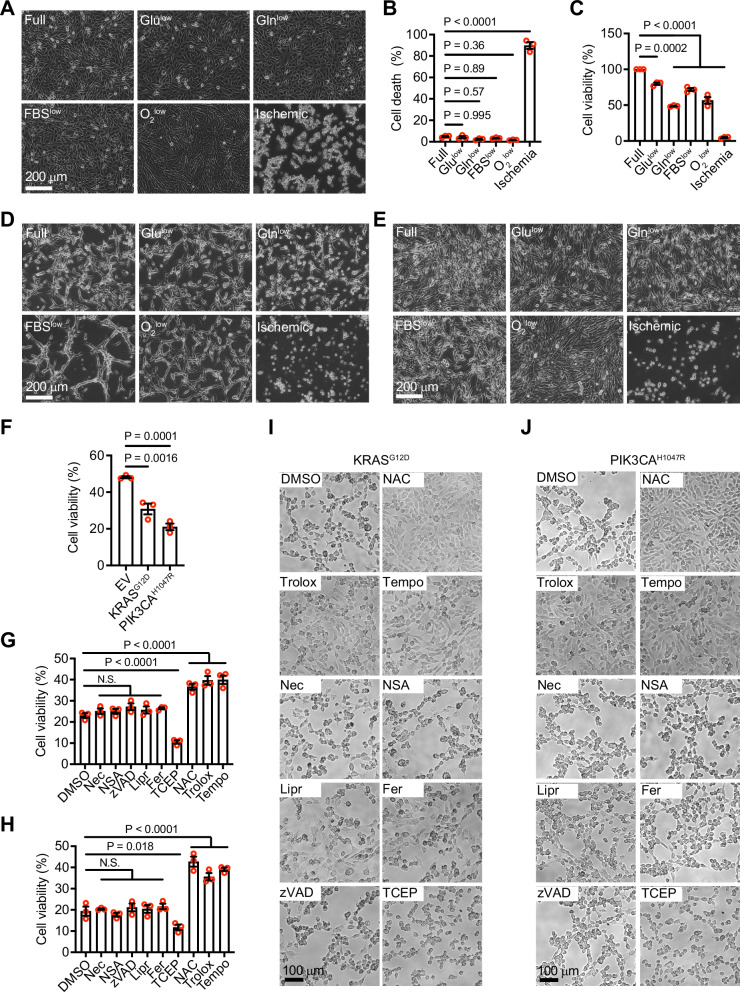
Activation of RAS and PI3K promotes ischemia-induced cell death. **A** Phase contrast images of LN229 cells cultured under different conditions as indicated for 24 h. **B** Cell death as measured by sytox green assay in LN229 cells cultured under different conditions for 24 h. Ordinary one-way ANOVA test. **C** Cell viability as measured by a crystal violet assay of LN229 cells cultured under different conditions for 24 h. Ordinary one-way ANOVA test. **D**, **E** Phase contrast images of U87MG (**D**) and U118MG (**E**) cells cultured under different conditions as indicated for 24 h. **F** Cell viability of LN229^vector^, LN229^KRAS(G12D)^, or LN229^PIK3CA(H1047R)^ cells cultured under the ischemic condition for 18 h. Ordinary one-way ANOVA test. Cell viability of LN229^KRAS(G12D)^ (**G**) or LN229^PIK3CA(H1047R)^ (**H**) cells cultured under the ischemic condition for 18 h with different cell death or ROS inhibitors. Ordinary one-way ANOVA test. Phase contrast images of LN229^KRAS(G12D)^ (**I**) or LN229^PIK3CA(H1047R)^ (**J**) cells cultured under the ischemic condition for 18 h with different cell death or ROS inhibitors.

To examine the response of GBM cells to ischemia, we cultured LN229 human GBM cells in conditions lacking oxygen and glucose [4]. Since GBM at the tumor center exhibits lower levels of glutamine compared to the tumor edge [15], we also partially deprived the media of glutamine. While deprivation of each component individually reduced cell proliferation (data not shown), the ability to induce cell death was much weaker than when all components were deprived together (Fig. 1A–C). For convenience, we have denoted this combinational deprivation condition as the ischemia-mimetic or ischemic condition hereafter. The enhanced cell death induced by the ischemic condition was also observed in two additional human GBM cell lines, U87MG and U118MG (Fig. 1D, E).

To examine the effect of RAS or PI3K activation on cell death under ischemic conditions, we employed patient-derived PIK3CA and RAS mutations. PIK3CA encodes the p110α catalytic subunit of PI3K and is mutated in 11% of GBM cases [6]. H1047R is one of the hotspot PIK3CA mutations known to drive GBM tumorigenesis [16]. The constitutively active RAS mutation, KRAS^G12D^, has also been observed in GBM. As expected, LN229 cells transduced with the constitutively active PIK3CA mutant (PIK3CA^H1047R^) or KRAS mutant (KRAS^G12D^), hereafter denoted LN229^PIK3CA(H1047R)^ or LN229^KRAS(G12D)^, respectively, showed increased phosphorylation of AKT or ERK1/2, respectively (Supplementary Fig. 1A, B). Next, we compared LN229^KRAS(G12D)^, LN229^PIK3CA(H1047R)^ and vector-transduced LN229 cells (LN229^vector^) cells under ischemic conditions. LN229^KRAS(G12D)^ and LN229^PIK3CA(H1047R)^ cells were more likely to die under the ischemic condition (Fig. 1F), suggesting that cell death can be enhanced by RAS or PI3K. To understand the nature of cell death under this condition, we employed a panel of small molecule inhibitors that specifically suppress certain types of regulated cell death. These inhibitors include z-VAD-FMK (zVAD; apoptosis), necrostatin-1 (Nec; necroptosis), necrosulfonamide (NSA; necroptosis), ferrostatin-1 (Fer; ferroptosis), liproxstatin-1 (Lipr; ferroptosis), tris-(2-carboxyethyl)-phosphine (TCEP; disulfidptosis), Trolox (ROS-induced cell death), Tempo (ROS-induced cell death) and N-acetyl-L-cysteine (NAC; ROS-induced cell death). Among these, NAC, Trolox, and Tempo increased the survival of both LN229^KRAS(G12D)^ and LN229^PIK3CA(H1047R)^ cells under the ischemic condition (Fig. 1G–J). This indicates that certain ROS are involved in cell death under the ischemic condition. Consistently, we observed increased ROS levels in LN229 cells cultured under the ischemic condition (Supplementary Fig. 1C, D). Therefore, the above results support that activation of RAS and PI3K enhances ROS-induced cell death in an ischemia-mimetic condition.

### ATF4 and IRE1α are required for ischemic tumor cell death

**Fig. 2:**
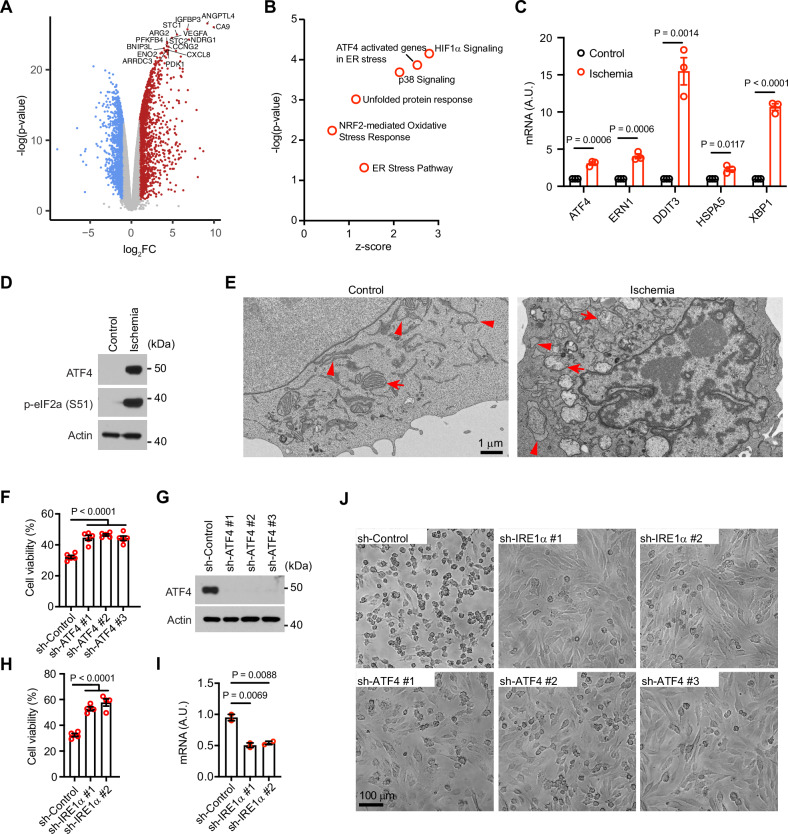
ATF4 and IRE1α are involved in ischemic tumor cell death. **A** Differentially expressed genes when comparing cells under the ischemic condition to those under the regular condition. **B** Ingenuity Pathway Analysis identified upregulated cellular stress pathways. **C** mRNA levels of each indicated gene in LN229 cells under regular (control) and ischemic conditions. **D** LN229 cells cultured under regular (control) or ischemic conditions were lysed and subjected to western blotting. **E** Representative TEM images from LN229 cells cultured under regular (control) or ischemic conditions. Arrows and arrowheads point to mitochondria and ER, respectively. **F**, **G** LN229 cells stably transduced by indicated shRNAs targeting ATF4 or a scrambled shRNA control were cultured in the ischemic condition for 18 h and subjected to crystal violet cell viability assay (**F**), or western blotting (**G**). **H**, **I** LN229 cells stably transduced by indicated shRNAs targeting IRE1α or a scrambled shRNA control were cultured in the ischemic condition for 18 h and subjected to crystal violet cell viability assay (**H**), or q-RT-PCR (**I**). **J** Phase contrast images of LN229 cells transduced by indicated shRNAs and cultured under ischemic conditions for 18 h. For **C**, Student’s *t* test. For **F**, **H** and **I** Ordinary one-way ANOVA test.

To further characterize the cellular response to ischemic conditions, we compared the gene expression profile of LN229 cells cultured under ischemic conditions to those cultured under regular conditions using RNA-sequencing (RNA-seq). We found that 1462 and 1126 genes were upregulated and downregulated (fold change (FC) $\ge$ 2, *p* < 0.05), respectively, in the ischemic cells compared to the control cells (Fig. 2A). Through Ingenuity Pathway Analysis of the differentially expressed genes, we found that HIF1α signaling was the most significantly activated pathway (Fig. 2B). In addition, NRF2-mediated oxidative stress response was also activated (Fig. 2B and Supplementary Fig. 2A). These observations are consistent with the hypoxic conditions and increased ROS levels observed in the ischemic cells (Fig. 1), indicating that our experimental setup is appropriate. Among the top activated cellular stress pathways, we observed the UPR to the endoplasmic reticulum (ER) stress and the p38 signaling pathways (Fig. 2B and Supplementary Fig. 2B–D).

To assess if these two signaling pathways are involved in ischemic cell death, we examined the UPR pathway first. Using q-RT-PCR, we confirmed that the expression of several UPR signaling genes, including *ATF4, ERN1, DDIT3, HSPA5*, and *XBP1*, increased in ischemic LN229 cells (Fig. 2C). Consistently, phosphorylation of eIF2a was markedly increased, accompanied by elevated expression of ATF4 (Fig. 2D). We then examined ER morphology using transmission electron microscopy (TEM). In contrast to the long and narrow ER observed in normal tumor cells, ER in ischemic cells appeared enlarged (Fig. 2E, arrowheads). Additionally, mitochondrial morphology was markedly altered, with fewer and swollen cristae compared to healthy tumor cells (Fig. 2E, arrows). These observations suggest that tumor cells experience both ER and oxidative stress under ischemic conditions. To assess whether UPR signaling is involved in ischemic cell death, we examined ATF4 and IRE1α, which were upregulated in ischemic cells (Fig. 2B–D) and are key components of the two major UPR regulatory signaling branches [17]. Using shRNA-mediated knockdown, we found that depletion of either ATF4 by three different shRNAs or IRE1α by two different shRNAs increased cell survival under ischemic conditions (Fig. 2F–J). Additionally, the role of IRE1α was further investigated using two small molecule inhibitors, 4μ8c and Z4P. Both LN229^KRAS(G12D)^ and LN229^PIK3CA(H1047R)^ cells exhibited a dose-dependent increase in survival under ischemic conditions when treated with either inhibitor (Supplementary Fig. 2E–J). Collectively, these results demonstrate that activated UPR regulators, including ATF4 and IRE1α, play a role in promoting cell death under ischemic conditions.

### p38-MK2 signaling is required for ischemic tumor cell death

**Fig. 3:**
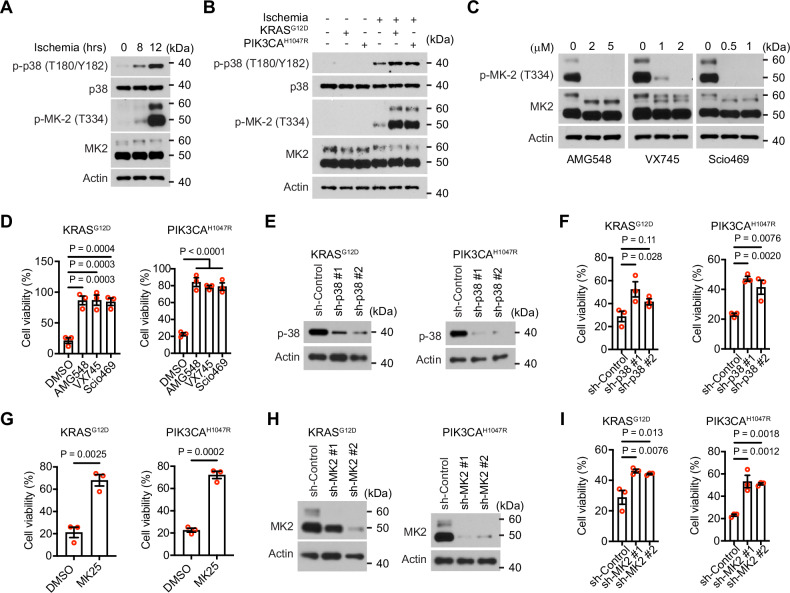
p38-MK2 signaling is required for ischemia-induced cell death. **A** LN229 cells were cultured under the ischemic condition for the indicated time and subjected to western blotting. **B** LN229^vector^, LN229^KRAS(G12D),^ or LN229^PIK3CA(H1047R)^ cells cultured under regular or ischemic conditions were subjected to western blotting. **C** LN229 cells treated by indicated p38 inhibitors or the DMSO control were subjected to western blotting. **D** Viability of LN229^KRAS(G12D)^ and LN229^PIK3CA(H1047R)^ cells treated with indicated p38 inhibitors (AMG548, 2 μM; VX745, 2 μM; Scio469, 1 μM) under ischemic conditions. Ordinary one-way ANOVA test. **E** LN229^KRAS(G12D)^ or LN229^PIK3CA(H1047R)^ cells stably transduced by indicated shRNAs targeting p38α or a scrambled shRNA control were subjected to western blotting. **F** Cells, as indicated in **E**, were cultured in the ischemic condition for 18 h and subjected to crystal violet cell viability assay. Ordinary one-way ANOVA test. **G** Viability of LN229^KRAS(G12D)^ and LN229^PIK3CA(H1047R)^ cells treated with DMSO or MK25 (10 μM) under ischemic conditions. Student’s *t* test. **H** LN229^KRAS(G12D)^ or LN229^PIK3CA(H1047R)^ cells stably transduced by indicated shRNAs targeting MK2 or a scrambled shRNA control were subjected to western blotting. **I** Cells, as indicated in **H**, were cultured in the ischemic condition for 18 h and subjected to crystal violet cell viability assay. Ordinary one-way ANOVA test.

Gene expression profiling in ischemic cells suggested that p38 signaling is activated (Fig. 2B and Supplementary Fig. 2C). To further test this notion, we examined the phosphorylation of p38α on Thr 180 and Tyr 182, and the phosphorylation of MK2 on Thr 334. LN229 cells cultured under the ischemic condition exhibited a temporal increase in the phosphorylation of p38α and MK2 (Fig. 3A). This phosphorylation was further enhanced in LN229^KRAS(G12D)^ and LN229^PIK3CA(H1047R)^ cells (Fig. 3B). To examine if p38 is involved in ischemic cell death, we first employed three small molecule inhibitors of p38α: AMG548, VX745, and Scio469. The efficacy of these inhibitors in suppressing p38α was confirmed by their ability to inhibit its downstream target MK2 phosphorylation (Fig. 3C). These inhibitors effectively increased the survival of ischemic LN229^KRAS(G12D)^ and LN229^PIK3CA(H1047R)^ cells (Fig. 3D). Additionally, the inhibitors increased the survival of U87MG cells under the ischemic condition (Supplementary Fig. 3A). To further investigate the role of p38, we used two shRNAs to deplete p38α (Fig. 3E). Knockdown of p38α in LN229^KRAS(G12D)^ and LN229^PIK3CA(H1047R)^ cells increased their survival, although not as effectively as the p38α inhibitors (Fig. 3F). We then examined whether MK2 plays a role in ischemic cell death. First, we found that a small molecule inhibitor of MK2, MK25, increased the viability of both ischemic LN229^KRAS(G12D)^ and LN229^PIK3CA(H1047R)^ cells (Fig. 3G). Second, silencing the expression of MK2 using two different shRNAs (Fig. 3H) also increased the survival of LN229^KRAS(G12D)^ and LN229^PIK3CA(H1047R)^ cells under the ischemic condition (Fig. 3I). Knockdown of p38α or MK2 also increased the survival of ischemic LN229 cells (Supplementary Fig. 3B, C). These results indicate that p38-MK2 signaling is involved in promoting ischemic cell death.

### The connection between p38-MK2 signaling and UPR regulators

**Fig. 4:**
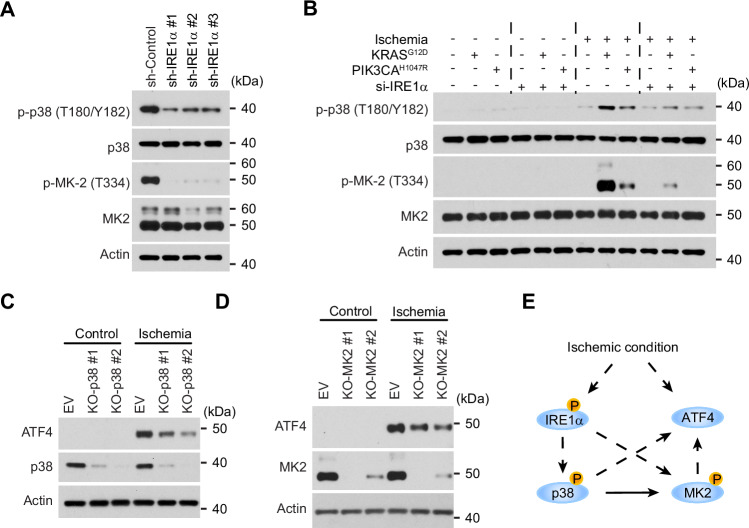
The connection between p38-MK2 signaling and UPR regulators. **A** LN229 cells stably transduced by indicated shRNAs targeting IRE1α or a scrambled shRNA control were cultured under the ischemic condition and subjected to western blotting. **B** LN229^vector^, LN229^KRAS(G12D)^ or LN229^PIK3CA(H1047R)^ cells transfected by a pool of four siRNA against IRE1α or scrambled siRNA and cultured under regular or ischemic conditions were subjected to western blotting. **C**, **D** LN229 cells stably transduced by indicated sgRNAs targeting p38 (**C**) or MK2 (**D**), or an empty vector (EV) were cultured under regular (control) or ischemic conditions and subjected to western blotting. **E** A diagram illustrating the signaling pathways activated under the ischemic condition.

Since both UPR regulators (ATF4 and IRE1α) and the p38-MK2 signaling are required for ischemic cell death, we thought to assess whether they have a certain causal relationship. While the knockdown of ATF4 in LN229 cells did not change the phosphorylation of MK2 and p38 (Supplementary Fig. 4A), silencing the expression of IRE1α effectively reduced the phosphorylation of both p38 and MK2 (Fig. 4A). In LN229^KRAS(G12D)^ and LN229^PIK3CA(H1047R)^ cells, silencing the expression of IRE1α through a pool of four siRNA can also reduce the phosphorylation of both p38 and MK2 (Fig. 4B). To examine the role of p38 and MK2, we knocked out each individually using CRISPR. Depletion of either p38 or MK2 can reduce the expression of ATF4 in ischemic cells (Fig. 4C, D). Therefore, these results indicate that the activation of p38-MK2 signaling under ischemic conditions depends on the UPR regulator IRE1α, and p38-MK2 signaling can also amplify the UPR signal by further increasing ATF4 expression (Fig. 4E).

### RAS- or PI3K-driven GBM progression requires p38

**Fig. 5:**
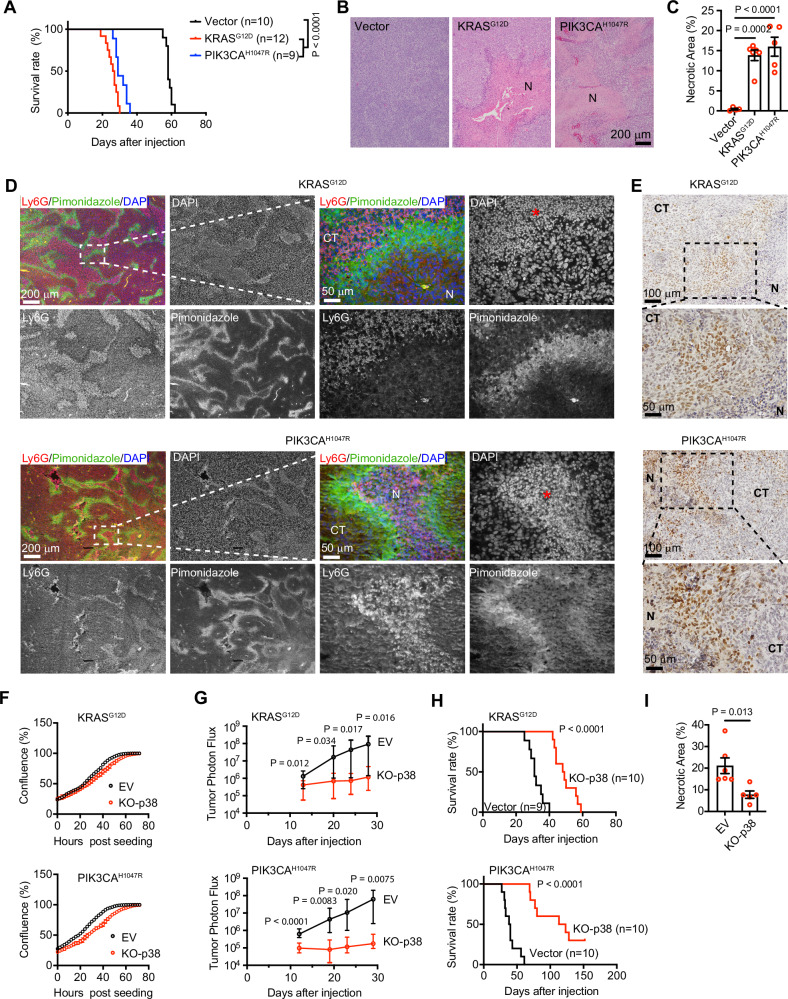
RAS or PI3K relies on p38 for GBM aggressiveness and generates tumor necrosis. **A** Kaplan–Meier survival curves comparing mice intracranially injected with LN229^vector^, LN229^KRAS(G12D)^, or LN229^PIK3CA(H1047R)^ cells. **B** H&E-stained tumor sections derived from LN229^vector^, LN229^KRAS(G12D)^ or LN229^PIK3CA(H1047R)^ terminal tumors. **C** Quantification of the necrotic area presented as the percentage of total tumor area in H&E-stained tumor sections from LN229^vector^, LN229^KRAS(G12D)^ or LN229^PIK3CA(H1047R)^ tumors. Ordinary one-way ANOVA test. **D** Immunofluorescent staining of mouse neutrophil marker, Ly6G, hypoxia marker, pimonidazole, and DAPI on paraffin-embedded sections of LN229^KRAS(G12D)^- or LN229^PIK3CA(H1047R)^-derived tumors upon reaching endpoints. **E** Chromogenic staining of p-MK2 on paraffin-embedded sections of LN229^KRAS(G12D)^- or LN229^PIK3CA(H1047R)^-derived endpoint tumors. In **B**, **D**, and **E**, N denotes tumor necrosis, and CT denotes cellular tumor. In **D**, the right images depict magnified views of the corresponding outlined areas from the left images. **F** Growth curve of LN229^KRAS(G12D)^ or LN229^PIK3CA(H1047R)^ cells cultured under regular conditions. (n = 3). **G** The size of tumors derived from LN229^KRAS(G12D)^ or LN229^PIK3CA(H1047R)^ cells transduced by an empty vector (EV) or p38 gRNA (KO-p38) was monitored using bioluminescence imaging. (n = 10 mice for each group, except n = 9 for EV of each tumor type at the last time point). Multiple unpaired t-test. **H** Kaplan-Meier survival curves comparing mice intracranially injected with p38α-depleted (KO-p38) LN229^KRAS(G12D)^ or LN229^PIK3CA(H1047R)^ cells. Log-rank test. **I** Quantification of the necrotic area presented as the percentage of total tumor area in H&E-stained tumor sections from LN229^KRAS(G12D)^ cells transduced by EV or KO-p38. Student’s *t* test.

The above observation that RAS and PI3K activation promotes tumor cell death under ischemic conditions led us to investigate whether their activation can promote tumor necrosis. Mice intracranially implanted with LN229^KRAS(G12D)^ or LN229^PIK3CA(H1047R)^ cells exhibited markedly shorter survival than those injected with LN229^vector^ cells (Fig. 5A). Histological studies found that LN229^KRAS(G12D)^ or LN229^PIK3CA(H1047R)^ tumors were much more heterogenous than LN229^vector^ tumors and contained large areas of necrosis, whereas LN229^vector^ tumors did not develop detectable necrosis (Fig. 5B, C). Since heterogeneity and extensive necrosis are features of GBM, this histological appearance suggests that RAS or PI3K hyperactivation changes the tumor microenvironment and promotes GBM progression.

To examine whether hypoxia is associated with tumor necrosis, we used the hypoxia probe, pimonidazole. Tumor cells surrounding necrotic areas (peri-necrotic zone or PNZ) showed the highest pimonidazole signals in both LN229^KRAS(G12D)^ and LN229^PIK3CA(H1047R)^ tumors (Fig. 5D), suggesting that these cells experienced the strongest hypoxia. Notably, within the tumor areas marked by high pimonidazole signals, we observed an increased density of cell nuclei (Fig. 5D, red asterisks). These nuclei appeared smaller than those of surrounding tumor cells, suggesting that they belong to different identities. Neutrophils are preferentially recruited to the necrotic tumors [18–21]. To examine whether these cells are neutrophils, we used a murine neutrophil marker, Ly6G [22]. A large proportion of these cells were marked by Ly6G (Fig. 5D), indicating neutrophils are recruited to the necrotic areas.

The above studies found that p38-MK2 signaling is activated in ischemic tumor cells (Figs. 2B, 3A, B, and Supplementary Fig. 2C). To examine whether the signaling is also activated in the PNZ of LN229^KRAS(G12D)^ or LN229^PIK3CA(H1047R)^ tumors, we performed immunohistochemistry using an antibody recognizing phosphorylated MK2. Tumor cells close to the necrotic areas showed more intense p-MK2 staining than the cellular tumor (CT) areas (Fig. 5E), suggesting hyperactive p38 in the PNZ of these tumors. To examine whether p38 is involved in tumor necrosis formation, we knocked out p38α via CRISPR in LN229^KRAS(G12D)^ and LN229^PIK3CA(H1047R)^ cells (Supplementary Fig. 4B). Depletion of p38α in these cells did not change the phosphorylation of ERK and AKT (Supplementary Fig. 4B), two major downstream effectors of RAS and PI3K; although it slightly reduced cell proliferation in a regular culture condition (Fig. 5F). Interestingly, p38α-depleted LN229^KRAS(G12D)^ or LN229^PIK3CA(H1047R)^ tumors grew markedly slower than control tumors (Fig. 5G). Mice bearing these p38α-depleted LN229^KRAS(G12D)^ or LN229^PIK3CA(H1047R)^ tumors exhibited markedly longer survival compared to those with control tumors (Fig. 5H). We then examined p38α-depleted LN229^KRAS(G12D)^ tumors and found that necrotic areas in these tumors were smaller than those in vector-transduced LN229^KRAS(G12D)^ tumors (Fig. 5I). These results indicate that p38 is essential in mediating the oncogenic effect driven by RAS and PI3K in GBM.

### UPR regulators and p38 are activated in patient GBM PNZ

**Fig. 6:**
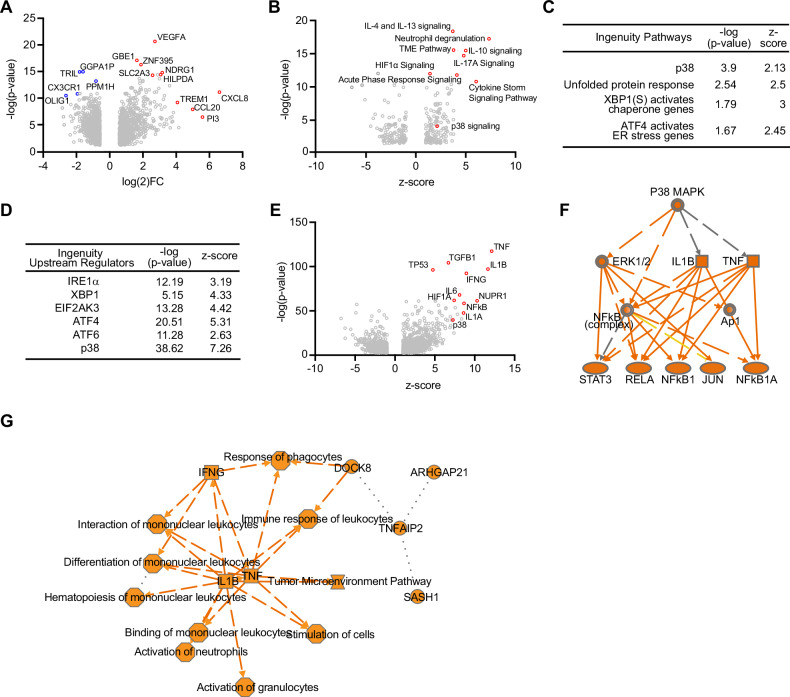
UPR regulators and p38 are activated in the PNZ of GBM. **A** Differentially expressed genes when comparing the tumor PNZ to the CT area. **B**, **C** Ingenuity Pathway Analysis identified differentially regulated signaling pathways (**B**), in which p38 and UPR-related pathways were shown in (**C**). Right-tailed Fisher’s exact test. **D** Ingenuity pathway analysis suggested that p38 and UPR regulators are activated in GBM PNZ. Right-tailed Fisher’s exact test. **E** Ingenuity Pathway Analysis predicted top-activated factors regulating tumor-promoting inflammatory factors. Right-tailed Fisher’s exact test. **F** The predicted downstream genes and their connections regulated by p38. **G** An integrated analysis of the upstream regulators, signaling pathways, and cellular processes to compare the tumor PNZ to the CT area.

To examine whether UPR regulators and p38 are also activated in tumor cells associated with necrotic areas in patient GBM, we employed a GBM dataset (Ivy Glioblastoma Atlas Project) [23], which comprises 26 tumor samples from the tumor PNZ and 111 samples from the CT area. 902 genes are upregulated, whereas 1179 genes are downregulated (FC$\,\ge \,$1.5, *p* < 0.05) in the PNZ compared to the CT region. Among these differentially expressed genes, the most significantly upregulated genes include well-characterized hypoxia-responding genes, such as *VEGFA, SLC2A3, HILPDA, CXCL8*, and *PI3* (Fig. 6A). Ingenuity Pathway Analysis of the differentially expressed genes confirmed that the HIF1α signaling is activated (z-score = 1.14, *p* = 1.62E−12) (Fig. 6B), which is consistent with the results showing increased signals of the hypoxia marker pimonidazole in the PNZ of the mouse models (Fig. 5D). The analysis also suggested that the p38 signaling (z-score = 2.13, *p* = 1.27E−4) and UPR-related pathways are more activated in the tumor PNZ than in the CT areas (Fig. 6B, C). Further analysis of the potential upstream regulators that could lead to the activation of these pathways suggested that p38 and several key UPR regulators are activated in the tumor PNZ (Fig. 6D). Therefore, the gene expression analysis indicated that activation of p38 and the UPR regulators are associated with the necrotic areas in GBM.

Our studies using the mouse GBM tumorigenesis models showed that p38 is required for tumor progression (Fig. 5F–I). To understand the underlying mechanism, we performed Ingenuity Molecule Activity Predictor Analysis with the above Ivy gene expression dataset. This analysis suggested that p38 could activate the downstream tumor-promoting inflammatory factors, such as TNF, IL1B, and NFkB, which are predicted to be the top-activated factors in the GBM PNZ (Fig. 6E, F). An integrated analysis of the upstream regulators, signaling pathways, and cellular processes related to the differentially expressed genes suggests that IL1B and TNF could be the central cytokines that stimulate multiple immune-related events, such as neutrophil and granulocyte activation, in the tumor microenvironment (Fig. 6G). Therefore, these cytokines downstream of p38 may mediate its tumor-promoting effect in GBM.

### The p38-MK2 signaling is responsible for the inflammatory gene expression program in the ischemic condition

**Fig. 7:**
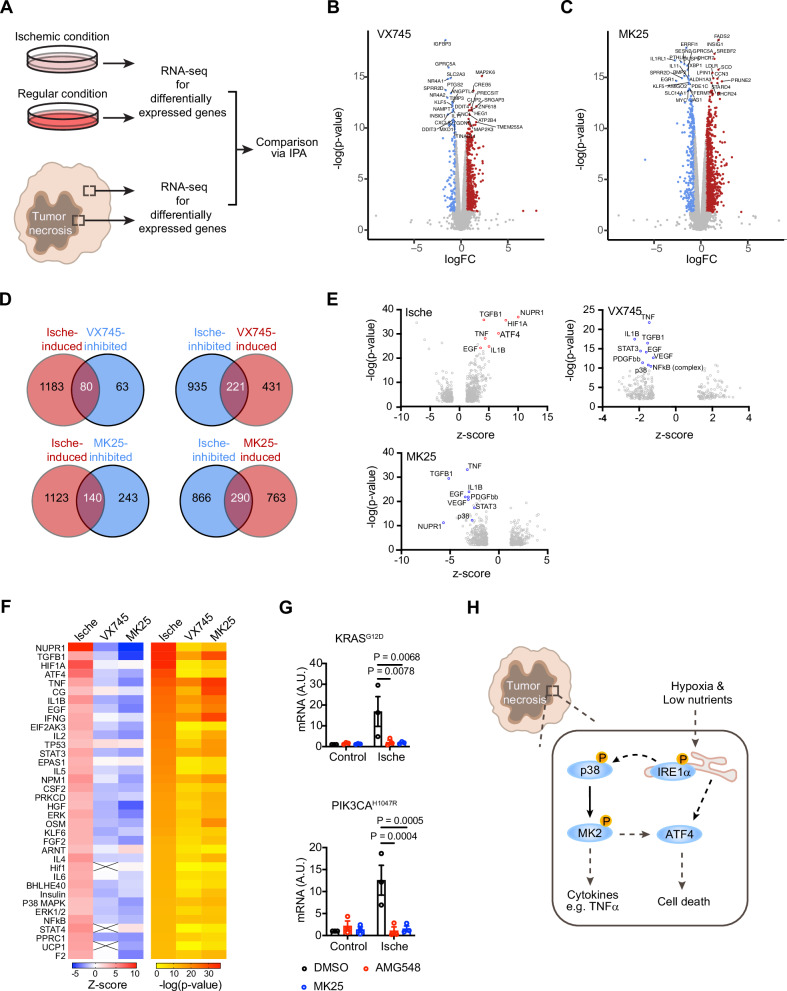
The p38-MK2 signaling pathway drives the inflammatory gene expression in response to ischemia. **A** A diagram describing the steps for analyzing and comparing differentially expressed genes in ischemic LN229 cells and the GBM PNZ relative to control LN229 cells and the GBM CT area, respectively. **B**, **C** Differentially expressed genes when comparing cells under the ischemic condition treated by VX745 (**B**) or MK25 (**C**) to those treated by a DMSO control. **D** Venn diagram showing overlapping genes in between indicated conditions. **E**, **F** Ingenuity Pathway Analysis identified upstream regulators under each indicated culture condition. **G** LN229^KRAS(G12D)^ or LN229^PIK3CA(H1047R)^ cells cultured under a regular (control) or the ischemic condition, treated by indicated inhibitors or DMSO were subjected to q-RT-PCR for TNFα mRNA. Ordinary two-way ANOVA test. **H** A diagram illustrating the involvement of the p38-MK2 and UPR signaling pathways in mediating ischemic stress-induced inflammatory response and cell death.

To examine whether the p38 signaling is involved in promoting the expression of inflammatory genes, we employed the in vitro ischemic condition. First, we used Ingenuity Pathway Analysis to compare the set of differentially expressed genes when LN229 cells were cultured under the ischemic or regular condition to those differentially expressed between tumor PNZ and CT regions (Fig. 7A). Multiple signaling pathways showed similar regulation between the ischemic cells and the PNZ of patient GBM (Supplementary Fig. 5A). Further analysis of the potential upstream regulators responsible for the gene expression changes revealed that the top-changed molecules also showed similar regulation between these two settings (Supplementary Fig. 5B). These results suggest that tumor cells under ischemic conditions recapitulate gene expression profiles of cells in the GBM PNZ. Then, we used the p38α inhibitor VX745 or the MK2 inhibitor MK25 in LN229 cells to inhibit p38α or MK2, respectively, and examined the gene expression programs under the ischemic condition. Compared to control cells treated by DMSO, gene expression in cells treated by VX745 or MK25 showed marked changes (Fig. 7B, C). Among genes significantly regulated by the ischemic condition (Ische-induced or -inhibited), expression changes of a set of these genes were reversed by VX745 or MK25 (Fig. 7D). Ingenuity Pathway Analysis of genes differentially expressed in VX745- or MK25-treated cells found that multiple activated molecules in ischemic cells, such as NUPR1, TGFB1, TNF, IL1B, and ATF4, were inhibited in VX745- or MK25-treated cells (Fig. 7E, F). Those downstream genes, such as *TNF, IL1B*, and *NFkB*, that were predicted to be activated by p38 in ischemic cells, were inhibited along with the inhibition of p38 signaling in these VX745- or MK25-treated cells (Supplementary Fig. 5C). To further test this notion, we examined the expression of TNFα by q-RT-PCR. In ischemic cells, expression of TNFα was markedly increased. However, treatment by the p38α inhibitor AMG548 or the MK2 inhibitor MK25 suppressed the increased expression of TNFα (Fig. 7G). These results indicate that the p38-MK2 signaling is responsible for the inflammatory gene expression response to the ischemic treatment.

## Discussion

This study identified the role of p38-MK2 signaling in promoting cell death and inflammatory effects in response to ischemia. When doing so, p38-MK2 signaling forms a positive feedback loop with UPR signaling. The signaling pathway can be further enhanced by the activation of RAS or PI3K, potentially driving the inflammatory response within necrotic tumors and promoting tumor progression (Fig. 7H).

It has been proposed that activation of the PI3K/AKT signaling pathway can protect tumor cells from apoptosis and channel them toward necrosis [3, 24]. These may explain why GBM, where the RTK/PI3K pathway is commonly activated, frequently forms necrosis [3, 24]. The observation that EGFR or mTORC1 activation promotes GBM cell death under hypoxic and partially glucose-deprived conditions, whereas PI3K/mTOR inhibition can prevent such cell death, supports this notion. However, later studies suggested that enhancing ATP depletion might be the underlying reason. In this study, using KRAS^(G12D)^ or PIK3CA^(H1047R)^, two mutations derived from GBM patients, we demonstrated that activation of the RAS/PI3K pathway promotes tumorigenesis and necrosis formation. We showed that activation of this pathway promotes cell death by activating ER stress and the p38-MK2 axis. These results may further help us to understand the role of PI3K/AKT activation in modulating the tumor microenvironment.

Various stresses and cytokines can activate p38 kinases [11]. It is commonly accepted that p38 helps cell survival during metabolic stress. However, sustained p38 activation can also increase cellular dependence on glucose and induce mitochondrial biogenesis and superoxide production, leading to MK2-mediated cell death [11]. In our study, we showed that p38 is activated under ischemic conditions, and its activation is further enhanced by RAS or PI3K. Both our findings and those of Canovas et al. [11] support a model where RAS/PI3K induces p38 activation, increasing cellular dependence on glucose. Under conditions of glucose, oxygen, and glutamine deprivation, cells die through an MK2-dependent mechanism. Furthermore, we found that UPR regulators, including IRE1α and ATF4, form a positive feedback loop with p38-MK2 in response to ischemic treatment. This suggests that UPR and p38-MK2 signaling form an intertwined regulatory network to regulate cellular responses to these conditions.

Necrosis in tumors can have tumor-promoting roles [1, 2]. By analyzing gene expression in GBM PNZ and tumor cells under ischemic conditions, our studies found that IL-4 and IL-13 signaling are the top-activated pathways (Fig. 6B). Signaling activated by these two cytokines can promote tumor growth by stimulating tumor cell proliferation and survival or modulating the immune system [25]. In addition, IL-10 and IL-17A, among the top activated pathways (Fig. 6B), have been shown to have tumor-promoting roles [26]. Further analysis indicated that TNF and IL1B are the two major cytokines shaping the microenvironment in the PNZ (Fig. 6G). This observation is consistent with their tumor-promoting functions [27]. In advanced cancers, p38α has been implicated in tumor promotion [11]. Our studies found that p38-MK2 signaling is responsible for activating the inflammatory transcriptional program under ischemic conditions and is essential for GBM tumorigenesis. This observation aligns with findings in breast cancer, where p38 regulates the expression of chemokines and cytokines in tumor cells, promoting the recruitment of pro-tumor myeloid populations [28]. Therefore, targeting the p38-MK2 axis may improve the efficacy of GBM treatments by preventing the pro-tumorigenic effects of necrosis-induced inflammation.

## Materials and methods

### Xenograft mouse tumor models

Six to eight-week-old female athymic nude mice (J:NU Strain Code: 007850, The Jackson Laboratory) were used for the GBM xenograft mouse models. Human GBM LN229 cells were transduced with retroviral vectors expressing KRAS^G12D^- or PIK3CA^H1047R^. For each mouse, 3 × 10^5^ cells were injected into the right hemisphere at 2 mm to the right of the bregma, 1 mm anterior to the coronal suture, and 3 mm into the brain using a Hamilton syringe. For tumor sample histology, whole brain tissue from tumor-bearing mice was fixed with 4% neutral-buffered formalin, embedded in paraffin, cut into 5 μm thick sections, and stained with hematoxylin & eosin (H&E). Tumor and central necrosis areas were manually traced and measured with ImageJ to quantify tumor area and necrosis-to-tumor ratio. To measure tumor growth using the in vivo imaging system (IVIS), the cells utilized for intracranial injection were transduced by the gene expressing firefly luciferase. Following induction of anesthesia, luciferin solution (GoldBio, LUCK, 15 mg/ml) was intraperitoneally injected into the mice at a dosage of 6 μl per gram of body weight. After 10 min, the mice were transferred to the IVIS chamber (Xenogen) and imaged for 1 minute at the highest sensitivity setting. Subsequent imaging sessions were conducted every 5 min until the signal diminished. The luciferase activity was measured as photons emitted per second using LivingImage software (Xenogen). To determine the necrosis-to-tumor ratio, areas of the tumor and necrosis were manually outlined and quantified using ImageJ. All animals were housed in a room with a 12-hour light/dark cycle, free access to a standard rodent diet, and water at ambient temperature maintained between 18-23 degrees Celsius and humidity between 40-60%.

### Ethics approval

All mouse experiments described in this study were carried out with the approval of the Penn State University Institutional Animal Care and Use Committee with the registration PROTO202001647, and in accordance with its guidelines.

### Cell culture

Human GBM cell lines LN229 (CRL-2611), U87MG (HTB-14), and U118MG (HTB-15) were purchased from ATCC. None of these cell lines were listed in the database of misidentified cell lines maintained by ICLAC and NCBI Biosample. These cell lines were not authenticated in this study. All cell lines were confirmed as *Mycoplasma* negative before experiments. All cells were cultured in Dulbecco’s modified Eagle’s medium (DMEM; Corning, 10-013-CV) supplemented with 10% fetal bovine serum (FBS; Gibco, 10437028) and 1% Antibiotic-Antimycotic Solution (Corning, 30-004-CI) in a humidified incubator at 37 °C with 5% CO_2_ unless otherwise indicated. To mimic ischemic conditions in vitro, a combination of low nutrients and hypoxia was used. Specifically, the day after seeding the cells, the medium was replaced with DMEM without glucose, L-glutamine, sodium pyruvate (Corning, 17-207-CV) supplemented with a low concentration of glucose (2 mM), glutamine (0.1 mM), and FBS (1%) as a low nutrient condition. Subsequently, the cell plates were transferred to Gas Pak pouches (BD, B260683) with BD Gas Pak EZ anaerobe with an indicator (BD, 260001) to establish the hypoxic (~1% O_2_) condition. To measure cell proliferation under regular culture conditions, 3000 cells were seeded into a 96-well plate. The following day, the plate was transferred to the IncuCyte system and incubated for 72 h. Time-lapse imaging was conducted every 2 h over three days. Using the IncuCyte software, a confluence (the ratio of cell area to image area) was determined, representing proliferation as a percentage of confluence.

### Antibodies

For western blotting, primary antibodies were used at dilutions ranging from 1/500 to 1/2000, while secondary antibodies were used at dilutions from 1/3000 to 1/10,000. For immunohistochemistry staining, primary antibodies were diluted from 1/50 to 1/200. The antibodies used for western blotting and immunohistochemistry staining included: ATF4 (Cell Signaling Technology, 11815), p-EIF2a (S51) (Cell Signaling Technology, 3398), β-actin (Cell Signaling Technology, 3700), p-p38 (T180/Y182) (Cell Signaling Technology, 4511), p38 (Cell Signaling Technology, 8690), p-MK2 (T334) (Cell Signaling Technology, 3007), MK2 (Cell Signaling Technology, 12155), p-ERK (Cell Signaling Technology, 4370), p-AKT(S473) (Cell Signaling Technology, 4060), anti-rabbit HRP-conjugated secondary antibody (Cell Signaling Technology, 7074S), anti-mouse HRP-conjugated antibody (Cell Signaling Technology, 7076S).

### Compounds

Reagents used to characterize cell death include: Necrostatin-1 30 µM (Cayman Chemicals, 11658), Necrosulfonamide (NSA) 1 µM (Cayman Chemicals, 20844), zVAD 20 µM (Med Chem Express, HY-16658), Liproxstatin-1 0.2 µM (Sigma, SML1414,), Ferrostatin-1 2 µM (Sigma, SML0583), TCEP 1 mM (Thermo Fisher, 77720), N-acetylcysteine 5 mM (Sigma, A7250), Trolox 100 µM (Sigma, 238813), 4-Hydroxy-TEMPO 50 µM (Sigma, 176141). Compounds targeting p38, MK2, and IRE1 include: AMG548 at 2 µM (Cayman Chemical, 34577), VX745 at 2 µM (Cayman Chemical, 18075), Scio469 at 1 µM (Cayman Chemical, 29484), MK25 at 10 µM (Cayman Chemical, 14399), 4µ8C at 1, 2, 5, 10 µM (Med Chem Express, HY-19707), Z4P at 1, 2, 5, 10 µM (Med Chem Express, HY-153773).

### Gene expression and silencing

pBabe-Kras (G12D), a gift from Channing Der (Addgene plasmid # 58902), and pBabe-PIK3CA (H1047R), a gift from Jean Zhao (Addgene plasmid # 12524) [29], were used to generate LN229 cells stably expressing KRAS (G12D) and PIK3CA (H1047R). Lentiviral vectors encoding shRNAs targeting ATF4 (TRCN0000013573, TRCN0000013574, TRCN0000013575), IRE1α (TRCN0000000528, TRCN0000000529), p38α (TRCN0000000509, TRCN0000000510), MK2 (TRCN0000002283, TRCN0000002285) were used to knockdown each gene from the indicated cells. All The cells expressing these plasmids were selected with 2 µg/ml puromycin. All shRNAs were obtained from the Penn State shRNA library core facility. siIRE1α SMART pool (Horizon Discovery, L-004951-02-0005) was transfected using DharmaFECT^TM^ transfection reagent (Horizon Discovery, T-2005-01). For p38 and MK2 knockout, sgRNA targeting p38 (#1: CTCAGATAAGAGAATTACAG, #2: ATAATGGCCGAGCTGTTGAC) and MK2 (#1: AATCATGAAGAGCATCGGTG, #2: AAAGTCAGTGAGTTTCAGGA) was cloned into LentiCRSPRv2GFP vector, a gift from David Feldser (Addgene plasmid # 82416) [30].

### Western blot

Cells were lysed in SDS lysis buffer containing 10 mM Tris (pH 7.5), 1% SDS, 50 mM NaF, and 1 mM Na3VO4 and heated at 98 °C for 5 min. The lysates were then passed through a 1 mL syringe three times and further incubated at 98 °C for 5 min. After centrifugation at 13,000 RPM for 2 min, the supernatant was used to measure protein concentration. The samples were combined with NuPAGE™ LDS Sample Buffer (4X) (Invitrogen, NP0008) containing 2-mercaptoethanol. The mixture was loaded onto 4–12% Bis-Tris SDS–PAGE gels (Invitrogen) for electrophoresis at 150 V for 90 min. Subsequently, proteins were transferred from the gel to the Immobilon-P membrane (Millipore) using a constant current of 250 mA for 3 h. The membranes were then incubated in blocking buffer (5% skim milk/TBST) for 1 hr at room temperature, followed by overnight incubation with primary antibodies diluted in blocking buffer (ranging from 5000X to 2000X) at 4 °C. The next day, the membranes were washed three times for 7 min each with TBST (Tris-buffered saline with Tween 20) and then incubated with secondary antibodies (diluted from 3000X to 20,000X) for 1 hr at room temperature. After washing three times for 7 min each, the membranes were subjected to chemiluminescence detection using an ECL kit (Pierce, 1856136).

### qPCR

Total RNA was extracted using the TRIzol (Invitrogen, 15596018) reagent protocol. Subsequently, cDNA was synthesized utilizing the iScript cDNA Synthesis kit (Bio-Rad, catalog number 1708891), followed by quantitative PCR (qPCR) conducted on a CFX96 Touch Real-Time PCR Detection System using SsoAdvanced Universal SYBR Green Supermix (Bio-Rad, catalog number 1725271). GAPDH served as an internal reference to normalize the input cDNA. The primer sequences used were: ATF4 (FP: CCCTTCACCTTCTTACAACCTC, RP: TGCCCAGCTCTAAACTAAAGGA); IRE1α (FP: CATCCCCATGCCGAAGTTCA, RP: CTGCTTCTCTCCGGTCAGGA); DDIT3 (FP: GAACGGCTCAAGCAGGAAATC, RP: TTCACCATTCGGTCAATCAGAG); HSPA5 (FP: CATCACGCCGTCCTATGTCG, RP: CGTCAAAGACCGTGTTCTCG); XBP1(FP: CCCTCCAGAACATCTCCCCAT, RP: ACATGACTGGGTCCAAGTTGT); TNFα (FP: GAGGCCAAGCCCTGGTATG, RP: CGGGCCGATTGATCTCAGC); GAPDH (FP: GGAGCGAGATCCCTCCAAAAT, RP: GGCTGTTGTCATACTTCTCATGG).

### Crystal Violet assay to determine cell viability

Cells were seeded in a 96-well plate at a density of 20,000 cells per well. The following day, cells were subjected to different experimental conditions: control (complete medium and normoxia) or ischemic mimic conditions with or without drug treatments. After 18 h of treatment, the medium was aspirated, and cells were gently washed once with DBPS to minimize disturbance. Cells were fixed with 4% paraformaldehyde (PFA) for 10 min at room temperature. A staining solution containing 1% Crystal Violet in 20% methanol was added (50 µl per well). After a 10-min incubation at room temperature, the wells were washed twice with 200 µl of dH2O and air-dried overnight. To solubilize the staining solution, 10% acetic acid was added to each well and incubated for 10 min at room temperature. Cell viability was assessed by measuring the optical density at 590 nm using a plate reader (BMG Labtech). Readings were obtained in duplicate for each sample. Percent survival was calculated by normalizing the blank-subtracted value of cells under ischemic conditions to that of cells under control conditions.

### Sytox green assay to determine cell death

After incubation under ischemic conditions, cells were collected using Accutase (ICT, 490007-741). The collected cells were incubated with 50 nM sytox green (Invitrogen, S7020) in DMEM for 30 min at 37 °C. Subsequently, the samples were washed twice with DPBS and analyzed using BD LSRFortessa flow cytometer (BD Biosciences). Sytox green-positive cells were quantified using FITC channel from single cell populations.

### Quantification of ROS using CM-H2DCFDA

Intracellular ROS levels were detected using CM-H2DCFDA (Invitrogen, C6827). 0.4 ×10E6 cells were seeded in 35-mm dishes and incubated under either control or ischemic conditions for 16 h. After treatment, cells were harvested using Accutase (ICT, 490007-741), washed once with DPBS, and incubated with 2 µM CM-H2DCFDA in DPBS for 10 min at 37 °C. FVS780 was then added to the samples, which were incubated for 5 min at 37 °C. Following centrifugation at 1000 RPM for 4 min, cells were incubated in DMEM (Corning, 10-013-CV) supplemented with 10% fetal bovine serum for 15 min at 37 °C and washed twice with DPBS. ROS levels were detected using a BD LSRFortessa (BD Biosciences) flow cytometer. Intracellular ROS levels were quantified from live cells (FVS780 negative).

### Immunofluorescent staining and chromogenic staining

For immunofluorescent staining, the paraffin-embedded tissue slides were sequentially immersed in xylene and ethanol (100%, 95%, 70%, and 50%) followed by a rinse with dH2O for deparaffinization and rehydration. Then, the slides were heated at a sub-boiling temperature in 10 mM sodium citrate buffer (pH=6.0) for antigen retrieval. After cooling, the slides were rinsed with dH2O and rehydrated using a wash buffer (0.1% triton X-100 in DPBS). Following this, the slides were incubated with blocking buffer (2.5% BSA, 0.05% Triton X-100 in PBS) at room temperature for 1 hr and then incubated with primary antibodies diluted in blocking buffer (1:50-1:100) overnight at 4 °C. The next day, sections were washed three times with wash buffer (0.1% Triton X-100 in DPBS) for 10 min each, followed by incubation with a secondary antibody diluted in the blocking buffer (1:100-1:200) for 1 h at room temperature. Sections were again washed three times for 10 min each and then incubated with DAPI for nuclear staining for 1 min. Finally, the sections were mounted in ProLong^TM^ Gold Antifade Mountant (Invitrogen, P10144). For chromogenic staining, following antigen retrieval, the slides were rinsed with dH2O and subsequently incubated in a wash buffer for 10 min. Then, they were incubated with a peroxidase suppressor (Thermo Scientific, 35000) and washed with wash buffer for 5 min. The slides were incubated in 5% normal goat serum in a wash buffer for 30 min. Next, the slides were incubated with avidin and biotin (Vector laboratories, SP-2001) for 15 min each. Staining was carried out using primary antibodies (1:50-1:100) at 4 °C overnight. The following day, after washing with wash buffer three times for 5 min each, the biotinylated goat anti-rabbit IgG antibody (Vectastain, BP-9100-50) was applied to the slides for 30 min. They were then washed with wash buffer three times for 5 min each and incubated with a mixture of reagents A and B (Thermo scientific, PK-6200) for 30 min. The slides were rinsed three times with wash buffer and stained with hematoxylin solution to visualize the nuclei. The slides were then mounted using VectaMount Express Mounting Medium (VectorLabs, H-5700-60).

### Transmission electron microscopy

LN229 cells were seeded on a 60 × 15 mm Permanox dish (Nalge Nunc International) and cultured under ischemic conditions the following day. Subsequently, the cells underwent two washes with warm PBS and were then fixed with Karnovsky fixative at room temperature. The samples were additionally fixed in 1% osmium tetroxide in 0.1 M sodium cacodylate buffer (pH 7.4) for 1 h. After fixation, the samples were dehydrated in a graduated ethanol series followed by acetone and embedded in LX-112 (Ladd Research, Williston, VT). Sections with a thickness of 60 nm were prepared, stained with uranyl acetate and lead citrate, and examined using a JEOL JEM1400 Transmission Electron Microscope (JEOL USA INC., Peabody, MA).

### Detection of hypoxic area in the tumor

Mice were given an intraperitoneal injection of 60 mg/kg Hypoxyprobe-1 (Hypoxyprobe, HP-100mg). After 1 h, the brain was collected. The paraffin-embedded brain sections were processed for deparaffinization, antigen retrieval, and blocking, as described above. Then, the slides were incubated with FITC-Mab antibody (Hypoxyprobe, FITC-Mab) at 4 °C overnight. The next day, the slides were washed with washing buffer three times for 5 min each and then stained with DAPI. Following this, the sections were mounted using ProLong™ Gold Antifade Mountant (Invitrogen, P10144).

### RNA-sequencing and data processing

RNA sequencing was performed on an Illumina NovaSeq for 100 cycles using pair-end according to the manufacturer’s instructions. Sequencing data were analyzed using Galaxy (https://usegalaxy.org/). Briefly, reads were trimmed and aligned to reference the human genome and annotation files (GRCh38, build 38) using HISAT2. Differences in gene expression were detected by edgeR. Ingenuity Pathway Analysis (Qiagen) was then used for signaling pathway analysis based on the differential expressed genes. For genes differentially expressed in between the tumor peri-necrotic zone and the cellular tumor region, the comparison was performed through the website of the Ivy Glioblastoma Atlas Project (https://glioblastoma.alleninstitute.org/). For Ingenuity Pathway Analysis, 1.5-fold and *P* < 0.05 were used as the cut-off for differentially expressed genes. Indirect relationships were chosen.

### Statistics and reproducibility

All statistical calculations and plotting were conducted using GraphPad Prism 10. In vitro experiments utilized data points representing the average of technical replicates obtained from independent experiments. For in vivo experiments, each data point represents an individual animal. All center values are presented as means, and error bars indicate the standard errors of the mean. The sample size and p-value are shown on each graph. No sample size calculation was performed. Samples sizes were chosen based on if the differences between groups are biologically meaningful and are statistically significant. No data points were excluded in our study. For cell experiments, all cells in each experiment were from the same pool of parental cells. All mice used in this study have the same genetic background (nude mice). Animals were from the same cohort. Animals were randomized in a blinded fashion when tumor cells from different groups were implanted. All animals were maintained in the same environment and handled by the same procedure. For data collected by objective instruments, such as plate readers, qPCR cyclers, microscopy software, flow cytometers, animal IVIS systems, and western blotting, the investigators were not blinded to group allocation during data collection. For animal studies, the investigators were not blinded to group allocation during data collection. Additionally, for all animal studies, randomization occurred in a blinded fashion. The variance is similar between the groups that are being statistically compared.

## Supplementary information

Supplementary figures

## Data Availability

The RNA-seq datasets from this study have been deposited to the Gene Expression Omnibus with the accession # GSE281549.
