# Supplementary material for: Involvement of p38 MAPK and MAPKAPK2 in promoting cell death and the inflammatory response to ischemic stress associated with necrotic glioblastoma
Source: Cell Death Dis. 2025 Jan 14;16(1):12. doi: 10.1038/s41419-025-07335-3 (PMC11729867; doi:10.1038/s41419-025-07335-3)
Supplement: Supplementary file 1 — Supplementary figures [file 41419_2025_7335_MOESM1_ESM.pdf]

Supplemental Figure 1. (A) $LN229^{vector}$ or $LN229^{KRAS(G12D)}$ cells were cultured under a regular condition and subjected to western blotting. (B) $LN229^{vector}$ or $LN229^{PIK3CA(H1047R)}$ cells were cultured under a regular condition and subjected to western blotting. (C) Relative MFI of ROS indicator CM-H2DCFDA in control or ischemic LN229 cells. Student's t-test (D) Representative flow cytometry histogram showing control or ischemic LN229 cells stained with CM-H2DCFDA.

Supplemental Figure 2. (A)-(D) Signature genes regulated by NRF2-mediated oxidative stress response signaling pathway (A), the unfolded protein response signaling pathway (B), the p38 signaling pathway (C), or ATF4-regulated signaling (D) showed differential expression in between the ischemic and regular conditions. (E) and (F) Cell viability of $LN229^{KRAS(G12D)}$ cells treated with IRE1α inhibitors 4μ8c (E) or Z4P (F) at indicated concentrations and cultured under the ischemic condition for 18 hours. Ordinary one-way ANOVA test. (G) Phase contrast images of $LN229^{KRAS(G12D)}$ cells treated by 4μ8c and Z4P and cultured under the ischemic condition for 18 hours. (H) and (I) Cell viability of $LN229^{PIK3CA(H1047R)}$ cells treated with IRE1α inhibitors 4μ8c (H) or Z4P (I) at indicated concentrations and cultured under the ischemic condition for 18 hours. Ordinary one-way ANOVA test. (J) Phase contrast images of $LN229^{PIK3CA(H1047R)}$ cells treated by 4μ8c and Z4P and cultured under the ischemic condition for 18 hours.

Supplemental Figure 3. (A) Viability of U87MG cells treated with indicated p38 inhibitors under ischemic conditions. Ordinary one-way ANOVA test. (B) and (C) LN229 cells stably transduced by indicated shRNAs targeting p38α (B), MK2 (C), or a scrambled shRNA control were cultured in the ischemic condition for 18 hours and subjected to crystal violet cell viability assay. Ordinary one-way ANOVA test.

Supplemental Figure 4. (A) LN229 cells stably transduced by indicated shRNAs targeting ATF4 or a scrambled shRNA control were cultured under the ischemic condition and subjected to western blotting. (B) $LN229^{KRAS(G12D)}$ or $LN229^{PIK3CA(H1047R)}$ cells transduced by an empty vector (EV) or p38 gRNA (KO-p38) were cultured under a regular condition and subjected to western blotting.

Supplemental Figure 5. (A) Comparison of top-changed signaling pathways in the GBM PNZ and ischemic tumor cells. (B) Comparison of top-changed upstream regulators in the GBM PNZ and ischemic tumor cells. (C) Status (activation/inhibition) of the predicted downstream genes and their connections regulated by p38 under indicated culture conditions.

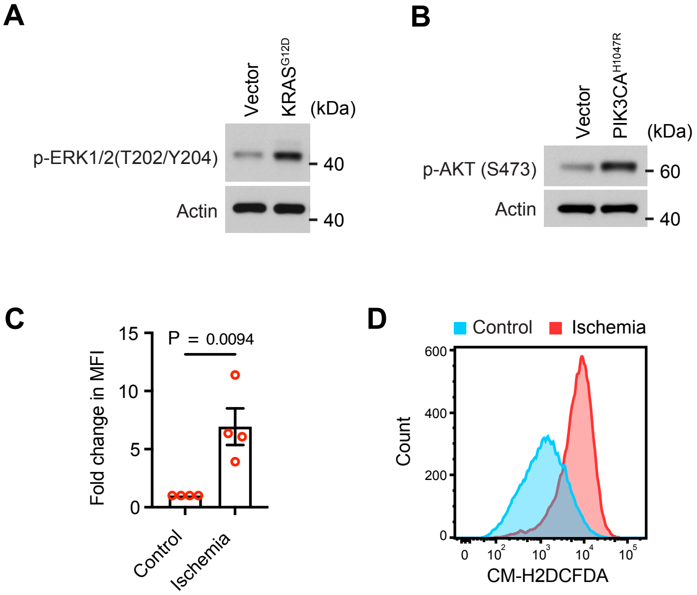
A
Vector
KRAS^{G12D}
(kDa)
p-ERK1/2(T202/Y204)
40
Actin
40
B
Vector
PIK3CA^{H1047R}
(kDa)
p-AKT (S473)
60
Actin
40
C
P = 0.0094
Fold change in MFI
15
10
5
0
Control
Ischemia
D
Control
Ischemia
Count
600
400
200
0
0
10^2
10^3
10^4
10^5
CM-H2DCFDA

Figure S1

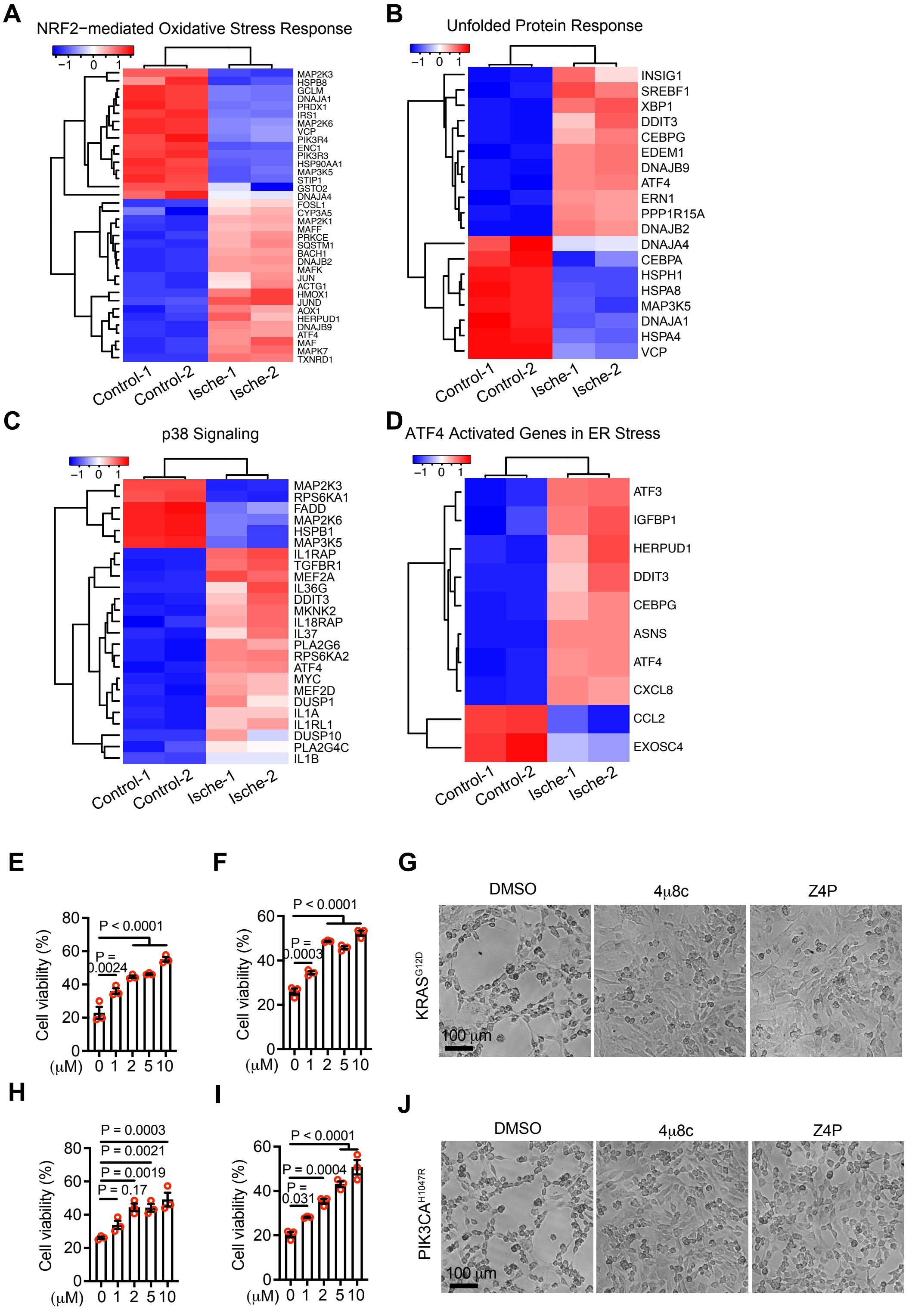
A
NRF2−mediated Oxidative Stress Response
B
Unfolded Protein Response
INSIG1
SREBF1
XBP1
DDIT3
CEBPG
EDEM1
DNAJB9
ATF4
ERN1
PPP1R15A
DNAJB2
DNAJA4
CEBPA
HSPH1
HSPA8
MAP3K5
DNAJA1
HSPA4
VCP
Control-1
Control-2
Ische-1
Ische-2
C
p38 Signaling
MAP2K3
RPS6KA1
FADD
MAP2K6
HSPB1
MAP3K5
IL1RAP
TGFBR1
MEF2A
IL36G
DDIT3
MKNK2
IL18RAP
IL37
PLA2G6
RPS6KA2
ATF4
MYC
MEF2D
DUSP1
IL1A
IL1RL1
DUSP10
PLA2G4C
IL1B
D
ATF4 Activated Genes in ER Stress
ATF3
IGFBP1
HERPUD1
DDIT3
CEBPG
ASNS
ATF4
CXCL8
CCL2
EXOSC4
E
F
G
DMSO
4μ8c
Z4P
KRAS^{G12D}
100 μm
H
I
J
PIK3CA^{H1047R}
Cell viability (%)
(μM)
P < 0.0001
P = 0.0024
P = 0.0003
P = 0.0021
P = 0.0019
P = 0.17
P = 0.0004
P = 0.031

Figure S2

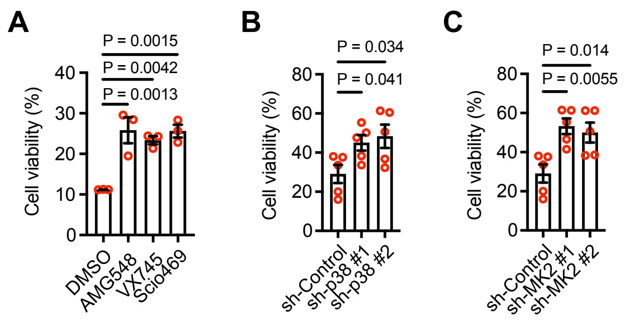
A
P = 0.0015
P = 0.0042
P = 0.0013
Cell viability (%)
DMSO
AMG548
VX745
Scio469
B
P = 0.034
P = 0.041
Cell viability (%)
sh-Control
sh-p38 #1
sh-p38 #2
C
P = 0.014
P = 0.0055
Cell viability (%)
sh-Control
sh-MK2 #1
sh-MK2 #2

Figure S3

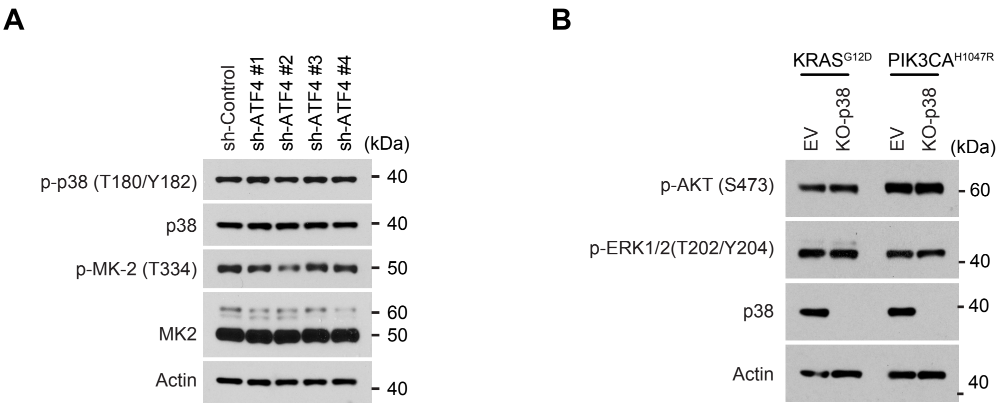
A
sh-Control
sh-ATF4 #1
sh-ATF4 #2
sh-ATF4 #3
sh-ATF4 #4
(kDa)
p-p38 (T180/Y182)
p38
p-MK-2 (T334)
MK2
Actin
40
40
50
60
50
40
B
KRAS^{G12D}
PIK3CA^{H1047R}
EV
KO-p38
EV
KO-p38
(kDa)
p-AKT (S473)
p-ERK1/2(T202/Y204)
p38
Actin
60
40
40
40

Figure S4

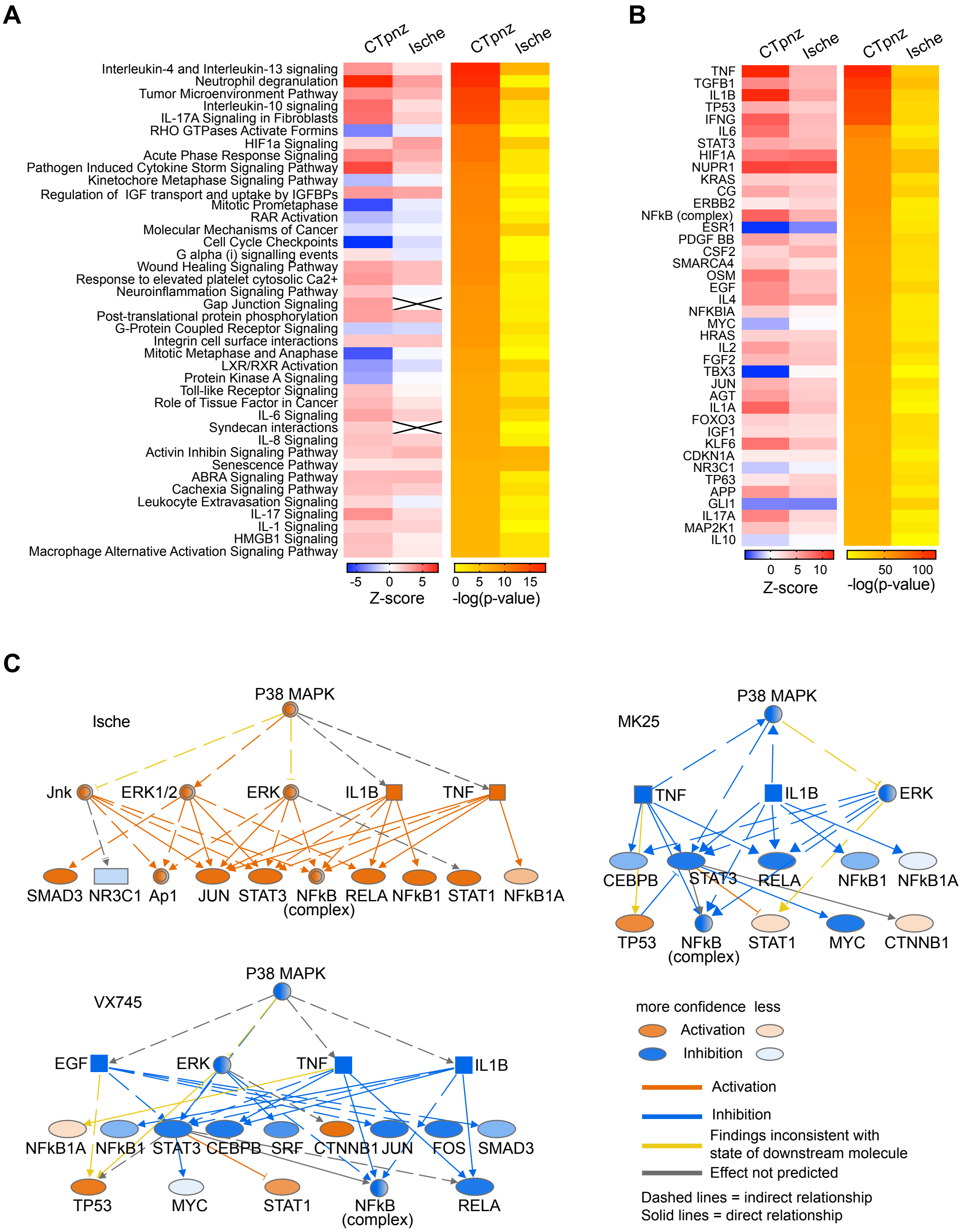
A
CTpnz
Ische
CTpnz
Ische
Interleukin-4 and Interleukin-13 signaling
Neutrophil degranulation
Tumor Microenvironment Pathway
Interleukin-10 signaling
IL-17A Signaling in Fibroblasts
RHO GTPases Activate Formins
HIF1a Signaling
Acute Phase Response Signaling
Pathogen Induced Cytokine Storm Signaling Pathway
Kinetochore Metaphase Signaling Pathway
Regulation of IGF transport and uptake by IGFBPs
Mitotic Prometaphase
RAR Activation
Molecular Mechanisms of Cancer
Cell Cycle Checkpoints
G alpha (i) signalling events
Wound Healing Signaling Pathway
Response to elevated platelet cytosolic Ca2+
Neuroinflammation Signaling Pathway
Gap Junction Signaling
Post-translational protein phosphorylation
G-Protein Coupled Receptor Signaling
Integrin cell surface interactions
Mitotic Metaphase and Anaphase
LXR/RXR Activation
Protein Kinase A Signaling
Toll-like Receptor Signaling
Role of Tissue Factor in Cancer
IL-6 Signaling
Syndecan interactions
IL-8 Signaling
Activin Inhibin Signaling Pathway
Senescence Pathway
ABRA Signaling Pathway
Cachexia Signaling Pathway
Leukocyte Extravasation Signaling
IL-17 Signaling
IL-1 Signaling
HMGB1 Signaling
Macrophage Alternative Activation Signaling Pathway
-5 0 5
Z-score
0 5 10 15
-log(p-value)
B
CTpnz
Ische
CTpnz
Ische
TNF
TGFB1
IL1B
TP53
IFNG
IL6
STAT3
HIF1A
NUPR1
KRAS
CG
ERBB2
NFkB (complex)
ESR1
PDGF BB
CSF2
SMARCA4
OSM
EGF
IL4
NFKBIA
MYC
HRAS
IL2
FGF2
TBX3
JUN
AGT
IL1A
FOXO3
IGF1
KLF6
CDKN1A
NR3C1
TP63
APP
GLI1
IL17A
MAP2K1
IL10
0 5 10
Z-score
50 100
-log(p-value)
C
Ische
P38 MAPK
Jnk
ERK1/2
ERK
IL1B
TNF
SMAD3
NR3C1
Ap1
JUN
STAT3
NFkB (complex)
RELA
NFkB1
STAT1
NFkB1A
MK25
P38 MAPK
TNF
IL1B
ERK
CEBPB
STAT3
RELA
NFkB1
NFkB1A
TP53
NFkB (complex)
STAT1
MYC
CTNNB1
VX745
P38 MAPK
EGF
ERK
TNF
IL1B
NFkB1A
NFkB1
STAT3
CEBPB
SRF
CTNNB1
JUN
FOS
SMAD3
TP53
MYC
STAT1
NFkB (complex)
RELA
more confidence less
Activation
Inhibition
Activation
Inhibition
Findings inconsistent with state of downstream molecule
Effect not predicted
Dashed lines = indirect relationship
Solid lines = direct relationship

Figure S5

Figure 2D

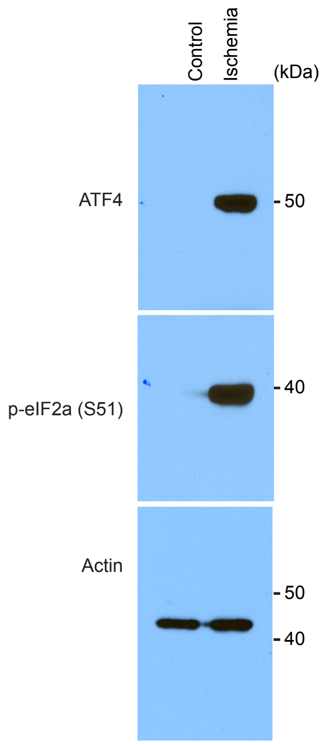

Figure 2G

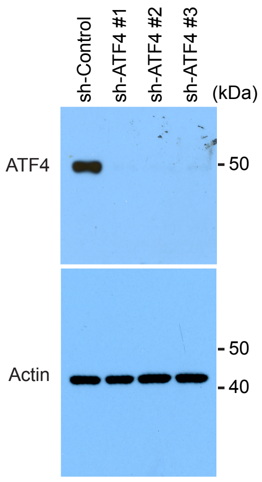

Figure 3A

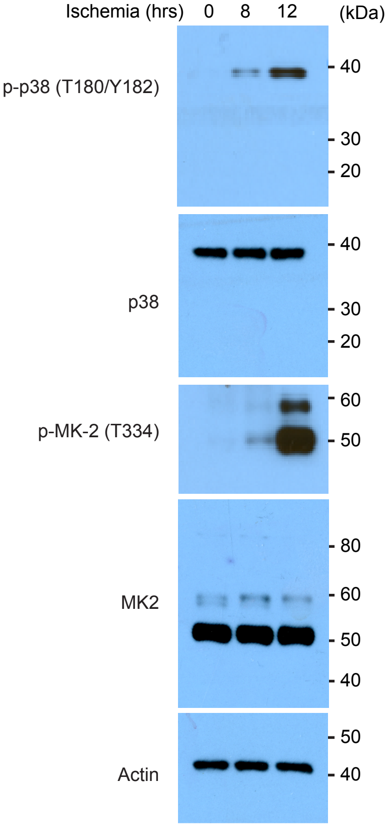

Figure 3B

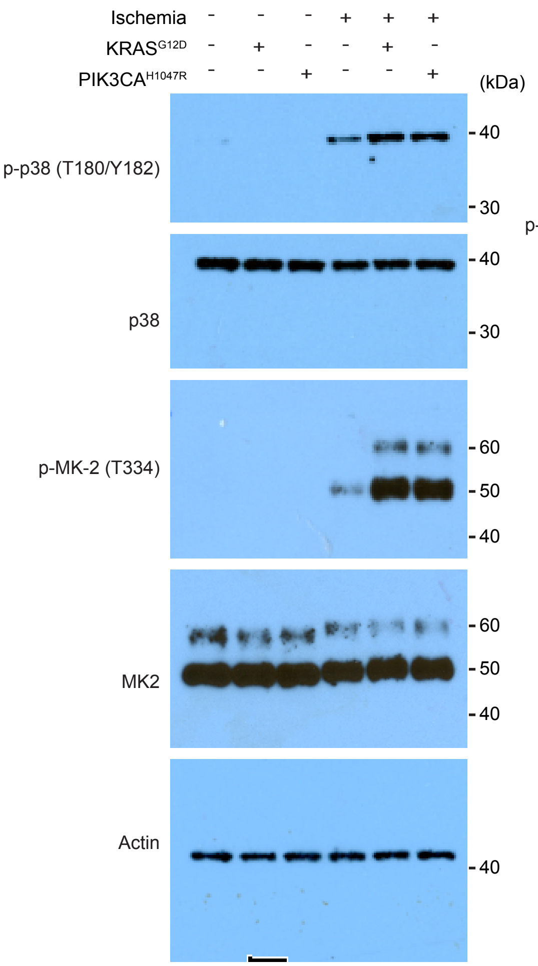

Figure 3C

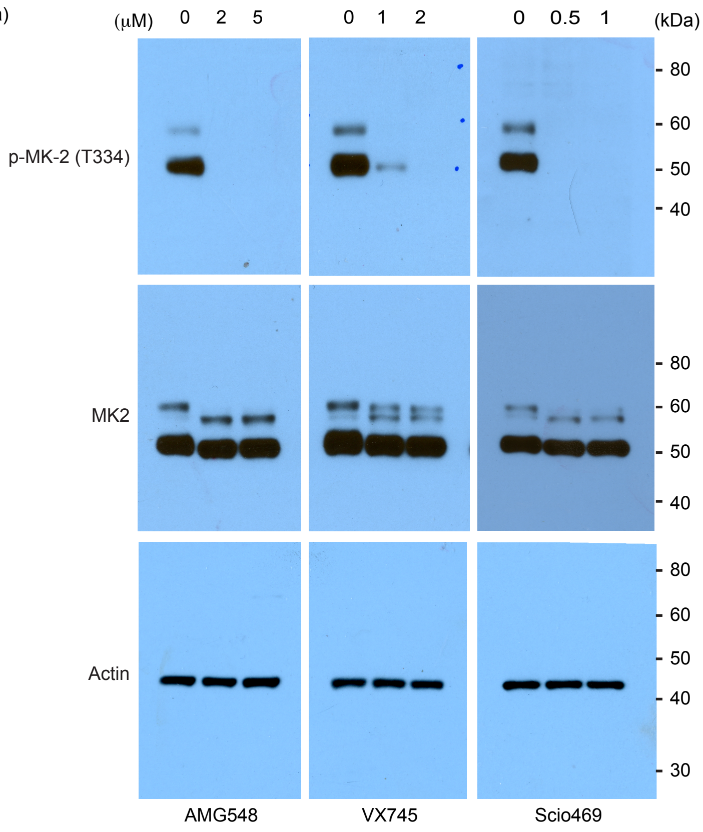

Figure 3E

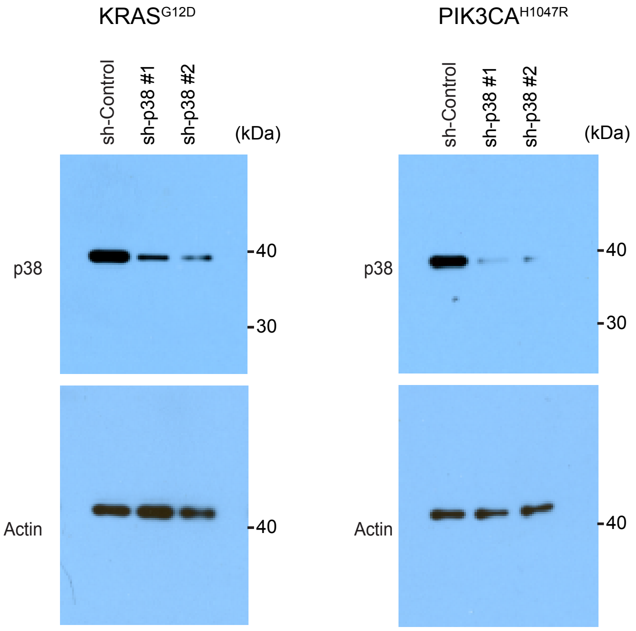

Figure 3H

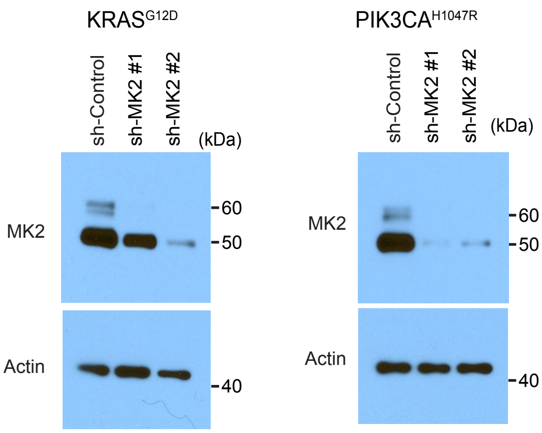

UncroppedWb_Figure 3

Figure 4A

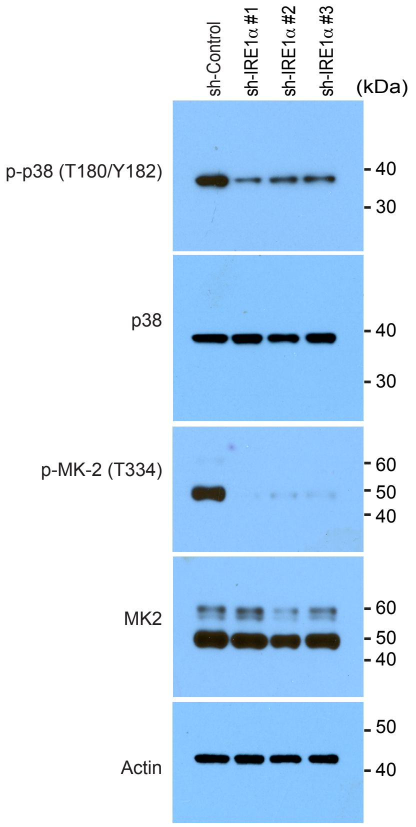

Figure 4B

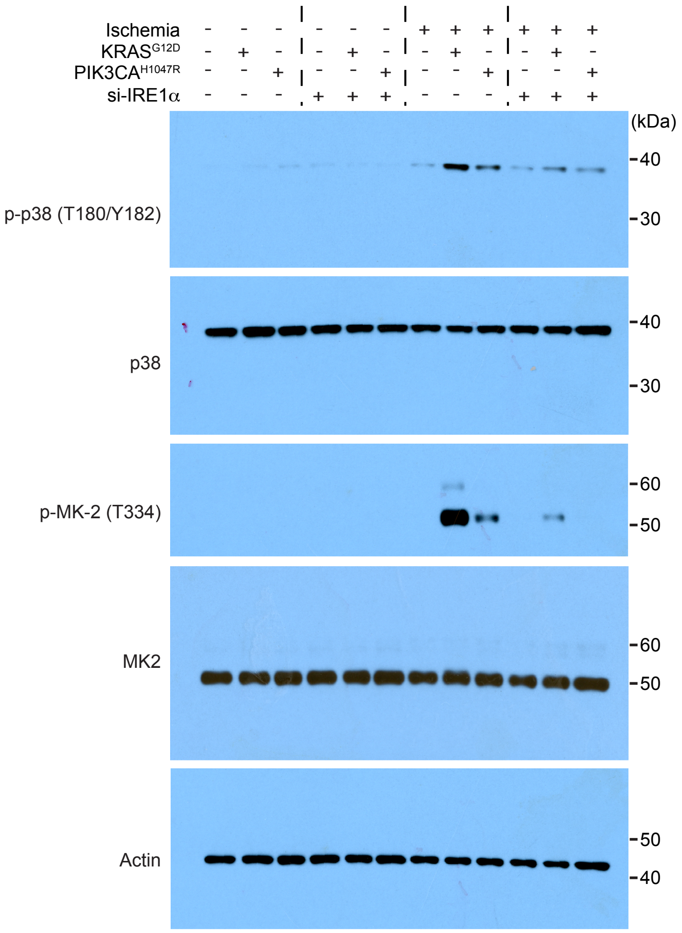

Figure 4C

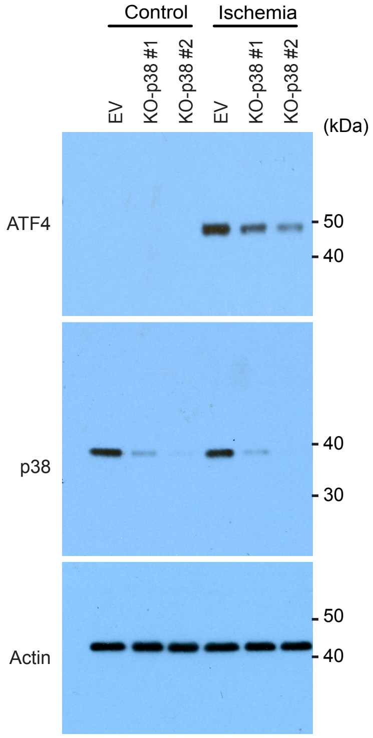

Figure 4D

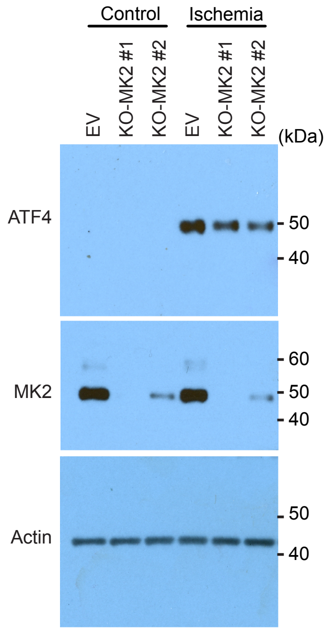

UncroppedWb_Figure 4

Figure S1A

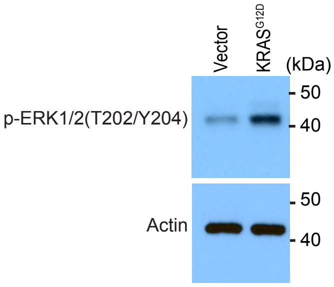

Figure S1B

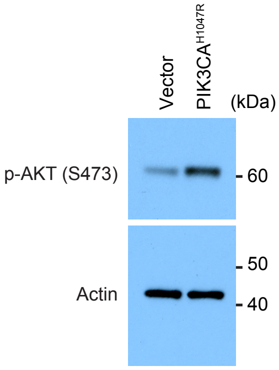

UncroppedWb_Figure S1

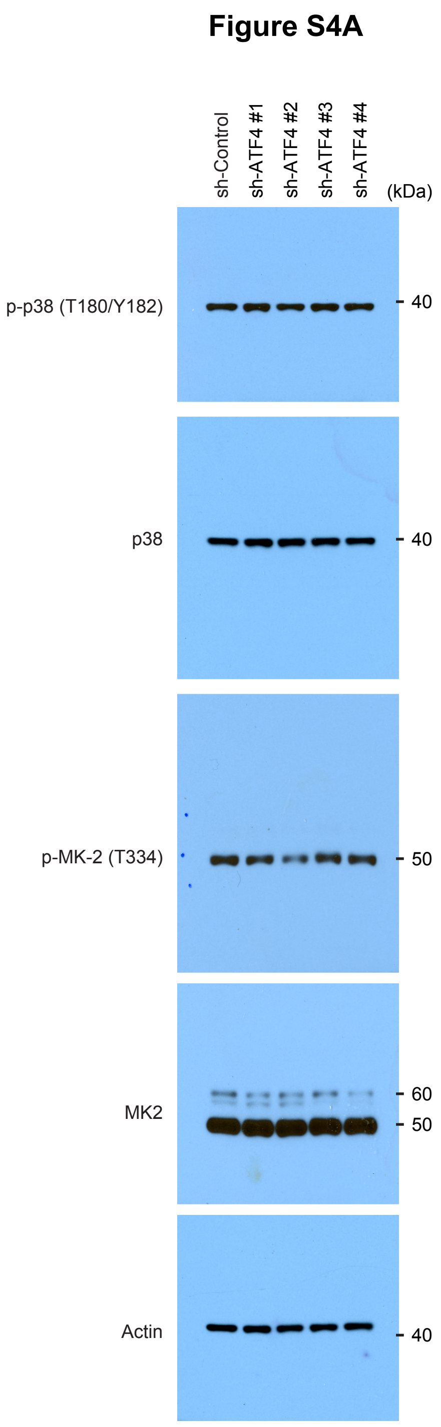

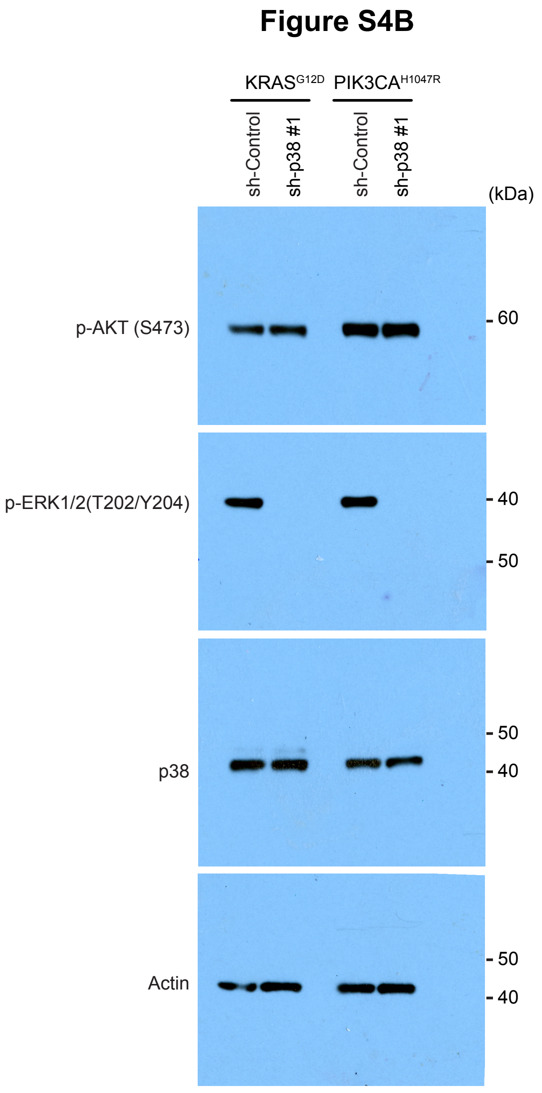

UncroppedWb_Figure S4
